# Supplementary figures and images for: Comparison of Different Machine Learning Algorithms for the Prediction of the Wheat Grain Filling Stage Using RGB Images (part 2 of 2)
Source: Plants (Basel). 2023 Nov 30;12(23):4043. doi: 10.3390/plants12234043 (PMC10708398; doi:10.3390/plants12234043)

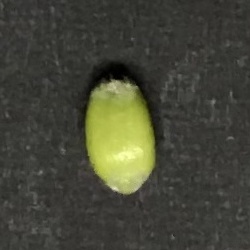

Supplement: Supplementary file 1 [file plants-12-04043-s001.zip › Figure S1-WheatGrain dataset/test/21/5-252-21-11.jpg]

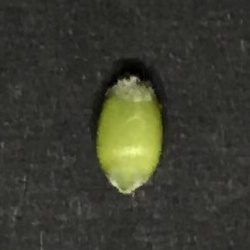

Supplement: Supplementary file 1 [file plants-12-04043-s001.zip › Figure S1-WheatGrain dataset/test/21/6-253-21-17.jpg]

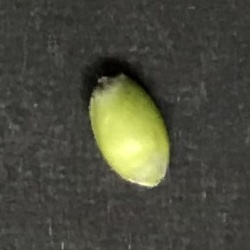

Supplement: Supplementary file 1 [file plants-12-04043-s001.zip › Figure S1-WheatGrain dataset/test/21/8-282-21-10.jpg]

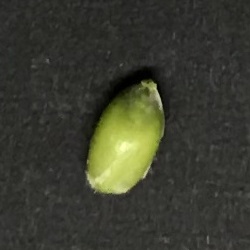

Supplement: Supplementary file 1 [file plants-12-04043-s001.zip › Figure S1-WheatGrain dataset/test/21/9-283-21-16.jpg]
