# Supplementary figures and images for: Comparison of Different Machine Learning Algorithms for the Prediction of the Wheat Grain Filling Stage Using RGB Images (part 1 of 2)
Source: Plants (Basel). 2023 Nov 30;12(23):4043. doi: 10.3390/plants12234043 (PMC10708398; doi:10.3390/plants12234043)

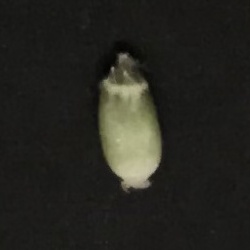

Supplement: Supplementary file 1 [file plants-12-04043-s001.zip › Figure S1-WheatGrain dataset/test/12/10-601-12-15.jpg]

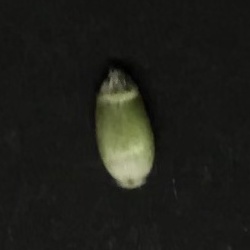

Supplement: Supplementary file 1 [file plants-12-04043-s001.zip › Figure S1-WheatGrain dataset/test/12/11-602-12-8.jpg]

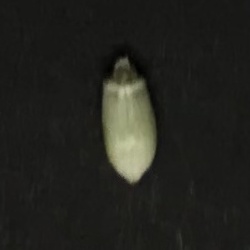

Supplement: Supplementary file 1 [file plants-12-04043-s001.zip › Figure S1-WheatGrain dataset/test/12/1-201-12-0.jpg]

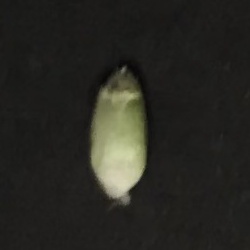

Supplement: Supplementary file 1 [file plants-12-04043-s001.zip › Figure S1-WheatGrain dataset/test/12/12-603-12-1.jpg]

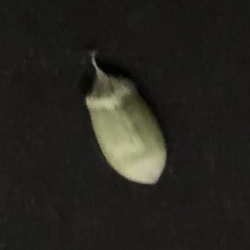

Supplement: Supplementary file 1 [file plants-12-04043-s001.zip › Figure S1-WheatGrain dataset/test/12/13-651-12-7.jpg]

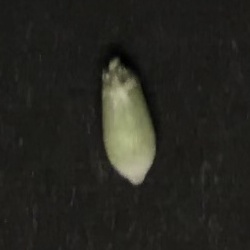

Supplement: Supplementary file 1 [file plants-12-04043-s001.zip › Figure S1-WheatGrain dataset/test/12/14-652-12-0.jpg]

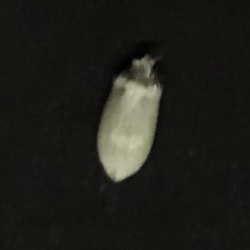

Supplement: Supplementary file 1 [file plants-12-04043-s001.zip › Figure S1-WheatGrain dataset/test/12/15-653-12-19.jpg]

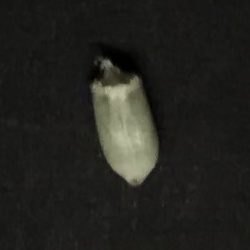

Supplement: Supplementary file 1 [file plants-12-04043-s001.zip › Figure S1-WheatGrain dataset/test/12/16-681-12-12.jpg]

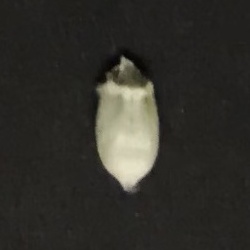

Supplement: Supplementary file 1 [file plants-12-04043-s001.zip › Figure S1-WheatGrain dataset/test/12/17-682-12-18.jpg]

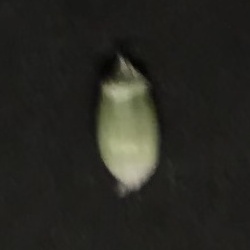

Supplement: Supplementary file 1 [file plants-12-04043-s001.zip › Figure S1-WheatGrain dataset/test/12/18-683-12-11.jpg]

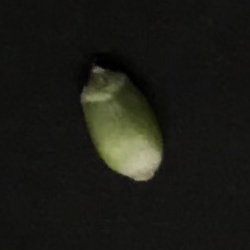

Supplement: Supplementary file 1 [file plants-12-04043-s001.zip › Figure S1-WheatGrain dataset/test/12/19-501-12-18.jpg]

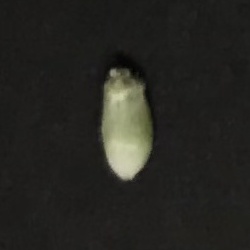

Supplement: Supplementary file 1 [file plants-12-04043-s001.zip › Figure S1-WheatGrain dataset/test/12/20-502-12-10.jpg]

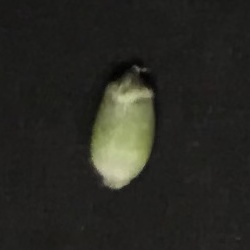

Supplement: Supplementary file 1 [file plants-12-04043-s001.zip › Figure S1-WheatGrain dataset/test/12/21-503-12-16.jpg]

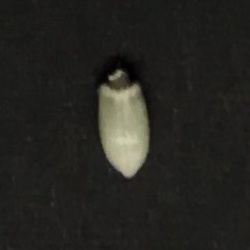

Supplement: Supplementary file 1 [file plants-12-04043-s001.zip › Figure S1-WheatGrain dataset/test/12/2-202-12-19.jpg]

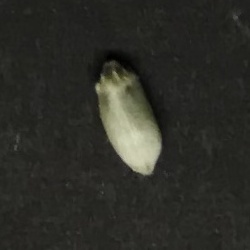

Supplement: Supplementary file 1 [file plants-12-04043-s001.zip › Figure S1-WheatGrain dataset/test/12/22-551-12-9.jpg]

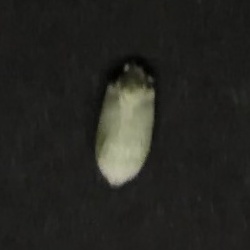

Supplement: Supplementary file 1 [file plants-12-04043-s001.zip › Figure S1-WheatGrain dataset/test/12/23-552-12-15.jpg]

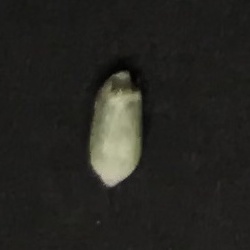

Supplement: Supplementary file 1 [file plants-12-04043-s001.zip › Figure S1-WheatGrain dataset/test/12/24-553-12-8.jpg]

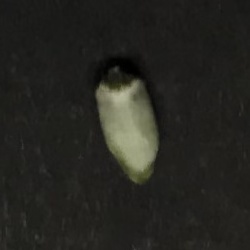

Supplement: Supplementary file 1 [file plants-12-04043-s001.zip › Figure S1-WheatGrain dataset/test/12/25-581-12-1.jpg]

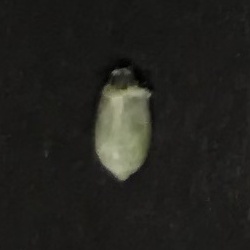

Supplement: Supplementary file 1 [file plants-12-04043-s001.zip › Figure S1-WheatGrain dataset/test/12/26-582-12-0.jpg]

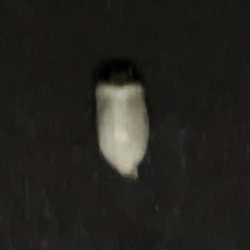

Supplement: Supplementary file 1 [file plants-12-04043-s001.zip › Figure S1-WheatGrain dataset/test/12/27-583-12-0.jpg]

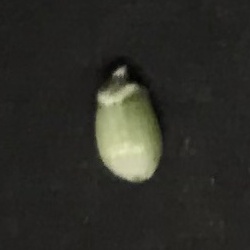

Supplement: Supplementary file 1 [file plants-12-04043-s001.zip › Figure S1-WheatGrain dataset/test/12/3-203-12-12.jpg]

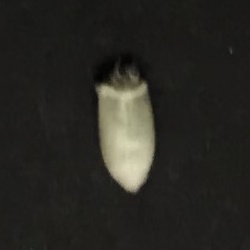

Supplement: Supplementary file 1 [file plants-12-04043-s001.zip › Figure S1-WheatGrain dataset/test/12/4-251-12-18.jpg]

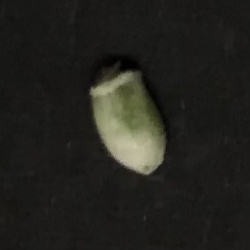

Supplement: Supplementary file 1 [file plants-12-04043-s001.zip › Figure S1-WheatGrain dataset/test/12/5-252-12-11.jpg]

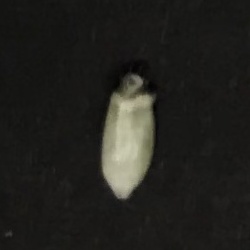

Supplement: Supplementary file 1 [file plants-12-04043-s001.zip › Figure S1-WheatGrain dataset/test/12/6-253-12-17.jpg]

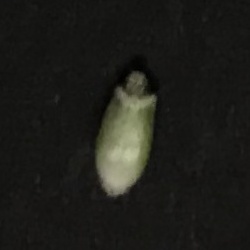

Supplement: Supplementary file 1 [file plants-12-04043-s001.zip › Figure S1-WheatGrain dataset/test/12/7-281-12-10.jpg]

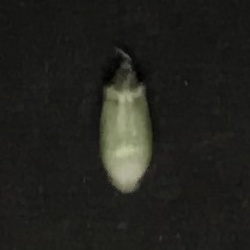

Supplement: Supplementary file 1 [file plants-12-04043-s001.zip › Figure S1-WheatGrain dataset/test/12/8-282-12-16.jpg]

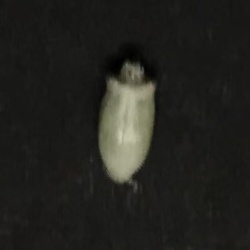

Supplement: Supplementary file 1 [file plants-12-04043-s001.zip › Figure S1-WheatGrain dataset/test/12/9-283-12-9.jpg]

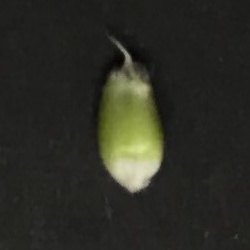

Supplement: Supplementary file 1 [file plants-12-04043-s001.zip › Figure S1-WheatGrain dataset/test/15/10-601-15-15.jpg]

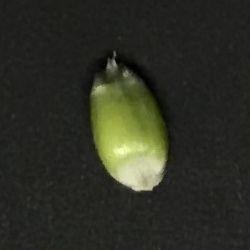

Supplement: Supplementary file 1 [file plants-12-04043-s001.zip › Figure S1-WheatGrain dataset/test/15/11-602-15-8.jpg]

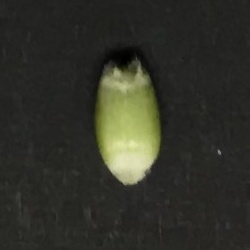

Supplement: Supplementary file 1 [file plants-12-04043-s001.zip › Figure S1-WheatGrain dataset/test/15/1-201-15-0.jpg]

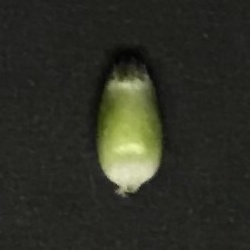

Supplement: Supplementary file 1 [file plants-12-04043-s001.zip › Figure S1-WheatGrain dataset/test/15/12-603-15-1.jpg]

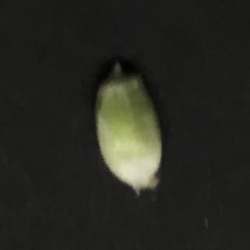

Supplement: Supplementary file 1 [file plants-12-04043-s001.zip › Figure S1-WheatGrain dataset/test/15/13-651-15-7.jpg]

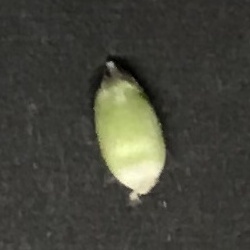

Supplement: Supplementary file 1 [file plants-12-04043-s001.zip › Figure S1-WheatGrain dataset/test/15/14-652-15-0.jpg]

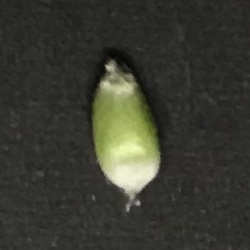

Supplement: Supplementary file 1 [file plants-12-04043-s001.zip › Figure S1-WheatGrain dataset/test/15/15-653-15-19.jpg]

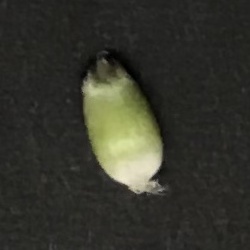

Supplement: Supplementary file 1 [file plants-12-04043-s001.zip › Figure S1-WheatGrain dataset/test/15/16-681-15-12.jpg]

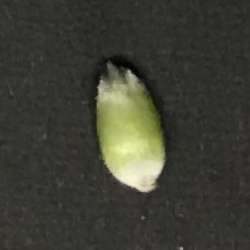

Supplement: Supplementary file 1 [file plants-12-04043-s001.zip › Figure S1-WheatGrain dataset/test/15/17-682-15-18.jpg]

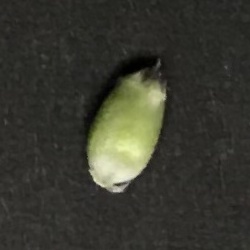

Supplement: Supplementary file 1 [file plants-12-04043-s001.zip › Figure S1-WheatGrain dataset/test/15/18-683-15-11.jpg]

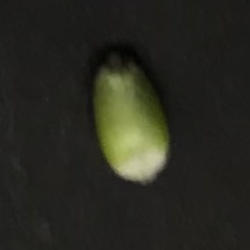

Supplement: Supplementary file 1 [file plants-12-04043-s001.zip › Figure S1-WheatGrain dataset/test/15/19-501-15-17.jpg]

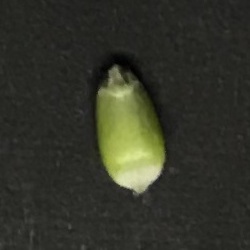

Supplement: Supplementary file 1 [file plants-12-04043-s001.zip › Figure S1-WheatGrain dataset/test/15/20-502-15-10.jpg]

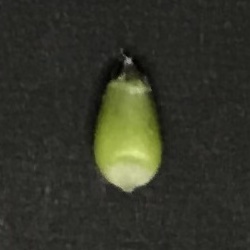

Supplement: Supplementary file 1 [file plants-12-04043-s001.zip › Figure S1-WheatGrain dataset/test/15/21-503-15-16.jpg]

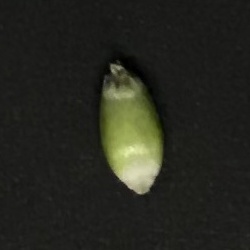

Supplement: Supplementary file 1 [file plants-12-04043-s001.zip › Figure S1-WheatGrain dataset/test/15/2-202-15-19.jpg]

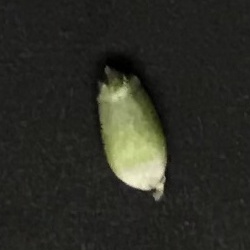

Supplement: Supplementary file 1 [file plants-12-04043-s001.zip › Figure S1-WheatGrain dataset/test/15/22-551-15-10.jpg]

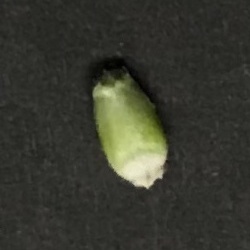

Supplement: Supplementary file 1 [file plants-12-04043-s001.zip › Figure S1-WheatGrain dataset/test/15/23-552-15-15.jpg]

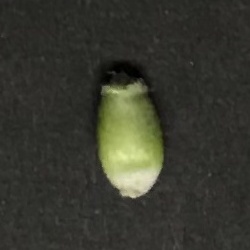

Supplement: Supplementary file 1 [file plants-12-04043-s001.zip › Figure S1-WheatGrain dataset/test/15/24-553-15-8.jpg]

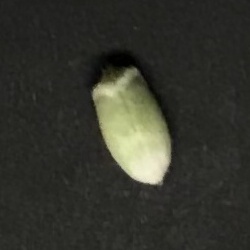

Supplement: Supplementary file 1 [file plants-12-04043-s001.zip › Figure S1-WheatGrain dataset/test/15/25-581-15-1.jpg]

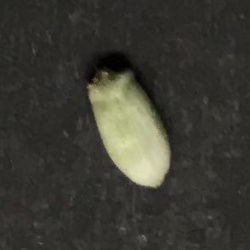

Supplement: Supplementary file 1 [file plants-12-04043-s001.zip › Figure S1-WheatGrain dataset/test/15/26-582-15-0.jpg]

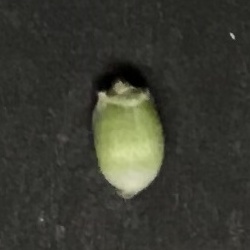

Supplement: Supplementary file 1 [file plants-12-04043-s001.zip › Figure S1-WheatGrain dataset/test/15/27-583-15-0.jpg]

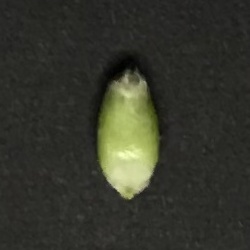

Supplement: Supplementary file 1 [file plants-12-04043-s001.zip › Figure S1-WheatGrain dataset/test/15/3-203-15-12.jpg]

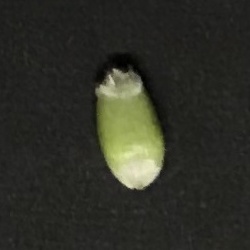

Supplement: Supplementary file 1 [file plants-12-04043-s001.zip › Figure S1-WheatGrain dataset/test/15/4-251-15-18.jpg]

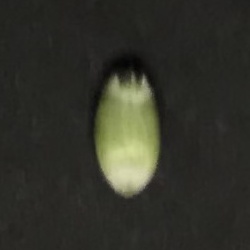

Supplement: Supplementary file 1 [file plants-12-04043-s001.zip › Figure S1-WheatGrain dataset/test/15/5-252-15-11.jpg]

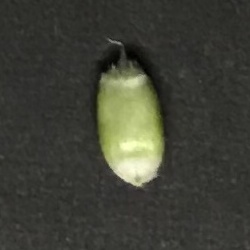

Supplement: Supplementary file 1 [file plants-12-04043-s001.zip › Figure S1-WheatGrain dataset/test/15/6-253-15-17.jpg]

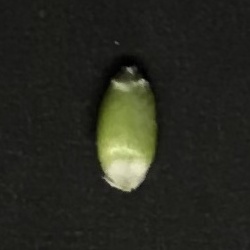

Supplement: Supplementary file 1 [file plants-12-04043-s001.zip › Figure S1-WheatGrain dataset/test/15/7-281-15-10.jpg]

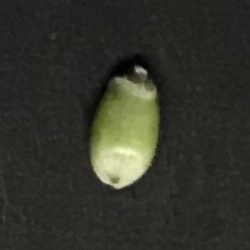

Supplement: Supplementary file 1 [file plants-12-04043-s001.zip › Figure S1-WheatGrain dataset/test/15/8-282-15-16.jpg]

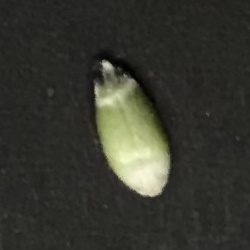

Supplement: Supplementary file 1 [file plants-12-04043-s001.zip › Figure S1-WheatGrain dataset/test/15/9-283-15-9.jpg]

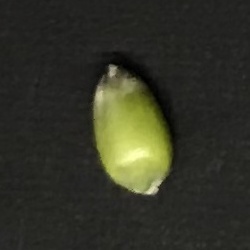

Supplement: Supplementary file 1 [file plants-12-04043-s001.zip › Figure S1-WheatGrain dataset/test/18/10-601-18-9.jpg]

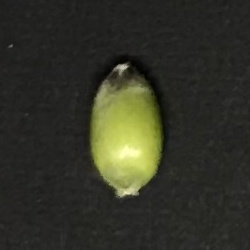

Supplement: Supplementary file 1 [file plants-12-04043-s001.zip › Figure S1-WheatGrain dataset/test/18/11-602-18-15.jpg]

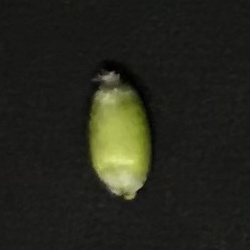

Supplement: Supplementary file 1 [file plants-12-04043-s001.zip › Figure S1-WheatGrain dataset/test/18/1-201-18-0.jpg]

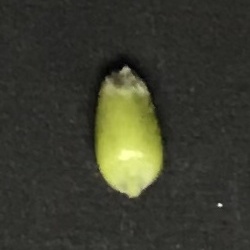

Supplement: Supplementary file 1 [file plants-12-04043-s001.zip › Figure S1-WheatGrain dataset/test/18/12-603-18-8.jpg]

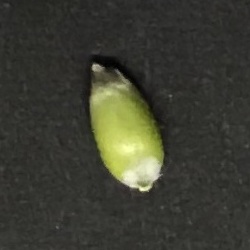

Supplement: Supplementary file 1 [file plants-12-04043-s001.zip › Figure S1-WheatGrain dataset/test/18/13-651-18-1.jpg]

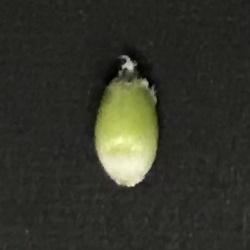

Supplement: Supplementary file 1 [file plants-12-04043-s001.zip › Figure S1-WheatGrain dataset/test/18/14-652-18-7.jpg]

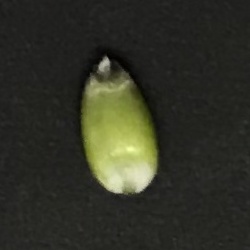

Supplement: Supplementary file 1 [file plants-12-04043-s001.zip › Figure S1-WheatGrain dataset/test/18/15-653-18-0.jpg]

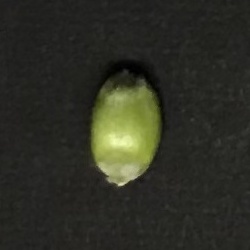

Supplement: Supplementary file 1 [file plants-12-04043-s001.zip › Figure S1-WheatGrain dataset/test/18/16-681-18-19.jpg]

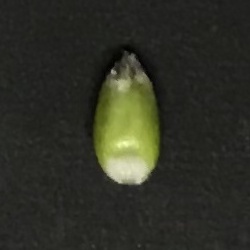

Supplement: Supplementary file 1 [file plants-12-04043-s001.zip › Figure S1-WheatGrain dataset/test/18/17-682-18-12.jpg]

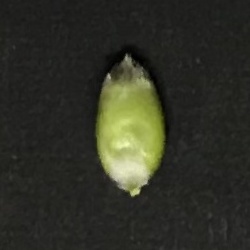

Supplement: Supplementary file 1 [file plants-12-04043-s001.zip › Figure S1-WheatGrain dataset/test/18/18-683-18-18.jpg]

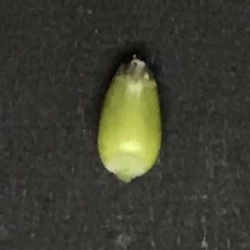

Supplement: Supplementary file 1 [file plants-12-04043-s001.zip › Figure S1-WheatGrain dataset/test/18/19-501-18-11.jpg]

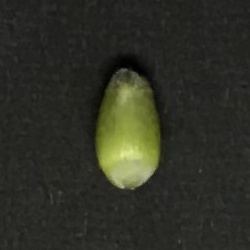

Supplement: Supplementary file 1 [file plants-12-04043-s001.zip › Figure S1-WheatGrain dataset/test/18/20-502-18-12.jpg]

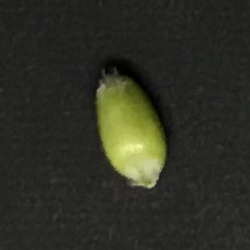

Supplement: Supplementary file 1 [file plants-12-04043-s001.zip › Figure S1-WheatGrain dataset/test/18/2-202-18-19.jpg]

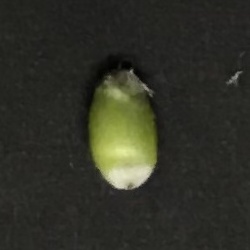

Supplement: Supplementary file 1 [file plants-12-04043-s001.zip › Figure S1-WheatGrain dataset/test/18/22-551-18-10.jpg]

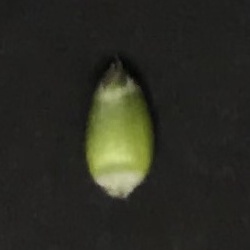

Supplement: Supplementary file 1 [file plants-12-04043-s001.zip › Figure S1-WheatGrain dataset/test/18/23-552-18-12.jpg]

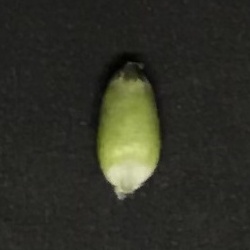

Supplement: Supplementary file 1 [file plants-12-04043-s001.zip › Figure S1-WheatGrain dataset/test/18/24-553-18-17.jpg]

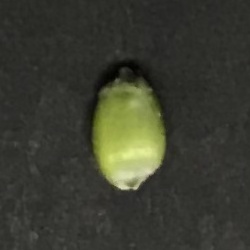

Supplement: Supplementary file 1 [file plants-12-04043-s001.zip › Figure S1-WheatGrain dataset/test/18/25-581-18-10.jpg]

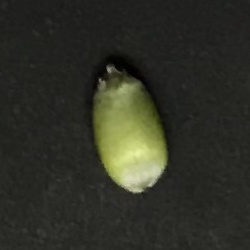

Supplement: Supplementary file 1 [file plants-12-04043-s001.zip › Figure S1-WheatGrain dataset/test/18/26-582-18-16.jpg]

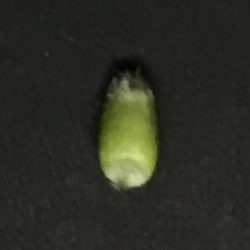

Supplement: Supplementary file 1 [file plants-12-04043-s001.zip › Figure S1-WheatGrain dataset/test/18/27-583-18-1.jpg]

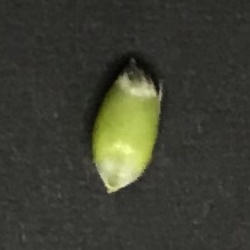

Supplement: Supplementary file 1 [file plants-12-04043-s001.zip › Figure S1-WheatGrain dataset/test/18/4-251-18-12.jpg]

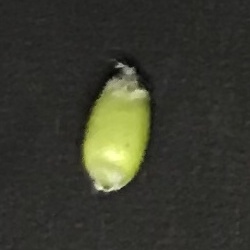

Supplement: Supplementary file 1 [file plants-12-04043-s001.zip › Figure S1-WheatGrain dataset/test/18/5-252-18-18.jpg]

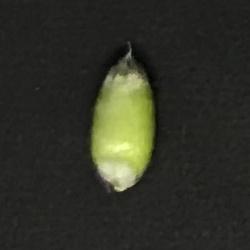

Supplement: Supplementary file 1 [file plants-12-04043-s001.zip › Figure S1-WheatGrain dataset/test/18/6-253-18-11.jpg]

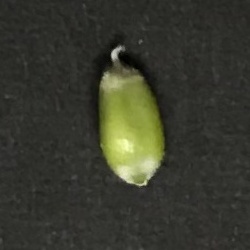

Supplement: Supplementary file 1 [file plants-12-04043-s001.zip › Figure S1-WheatGrain dataset/test/18/7-281-18-17.jpg]

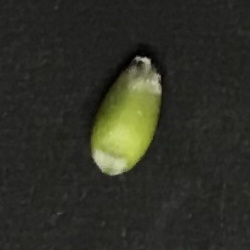

Supplement: Supplementary file 1 [file plants-12-04043-s001.zip › Figure S1-WheatGrain dataset/test/18/8-282-18-10.jpg]

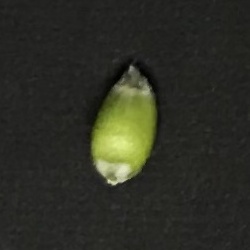

Supplement: Supplementary file 1 [file plants-12-04043-s001.zip › Figure S1-WheatGrain dataset/test/18/9-283-18-17.jpg]

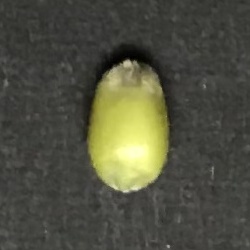

Supplement: Supplementary file 1 [file plants-12-04043-s001.zip › Figure S1-WheatGrain dataset/test/21/10-601-21-9.jpg]

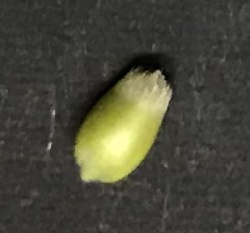

Supplement: Supplementary file 1 [file plants-12-04043-s001.zip › Figure S1-WheatGrain dataset/test/21/11-602-21-15.jpg]

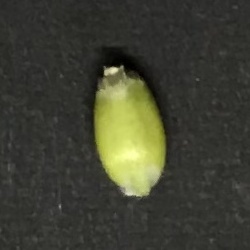

Supplement: Supplementary file 1 [file plants-12-04043-s001.zip › Figure S1-WheatGrain dataset/test/21/1-201-21-0.jpg]

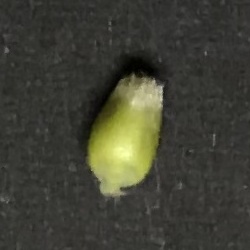

Supplement: Supplementary file 1 [file plants-12-04043-s001.zip › Figure S1-WheatGrain dataset/test/21/12-603-21-8.jpg]

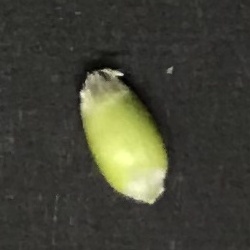

Supplement: Supplementary file 1 [file plants-12-04043-s001.zip › Figure S1-WheatGrain dataset/test/21/13-651-21-1.jpg]

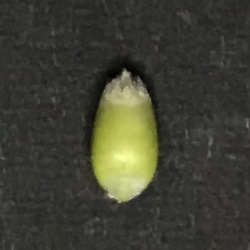

Supplement: Supplementary file 1 [file plants-12-04043-s001.zip › Figure S1-WheatGrain dataset/test/21/14-652-21-7.jpg]

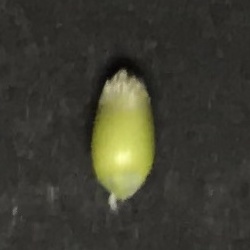

Supplement: Supplementary file 1 [file plants-12-04043-s001.zip › Figure S1-WheatGrain dataset/test/21/15-653-21-0.jpg]

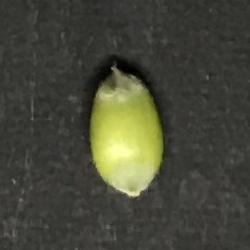

Supplement: Supplementary file 1 [file plants-12-04043-s001.zip › Figure S1-WheatGrain dataset/test/21/16-681-21-19.jpg]

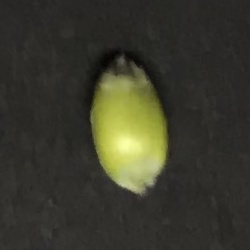

Supplement: Supplementary file 1 [file plants-12-04043-s001.zip › Figure S1-WheatGrain dataset/test/21/17-682-21-12.jpg]

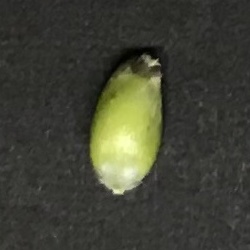

Supplement: Supplementary file 1 [file plants-12-04043-s001.zip › Figure S1-WheatGrain dataset/test/21/18-683-21-18.jpg]

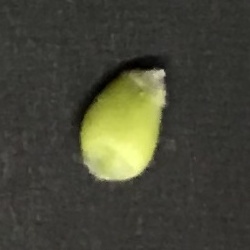

Supplement: Supplementary file 1 [file plants-12-04043-s001.zip › Figure S1-WheatGrain dataset/test/21/19-501-21-11.jpg]

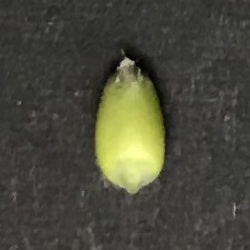

Supplement: Supplementary file 1 [file plants-12-04043-s001.zip › Figure S1-WheatGrain dataset/test/21/21-503-21-11.jpg]

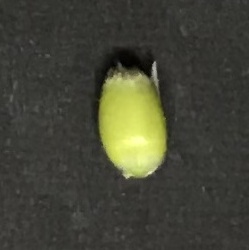

Supplement: Supplementary file 1 [file plants-12-04043-s001.zip › Figure S1-WheatGrain dataset/test/21/2-202-21-19.jpg]

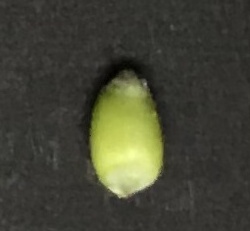

Supplement: Supplementary file 1 [file plants-12-04043-s001.zip › Figure S1-WheatGrain dataset/test/21/22-551-21-10.jpg]

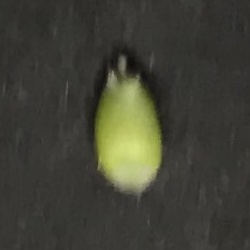

Supplement: Supplementary file 1 [file plants-12-04043-s001.zip › Figure S1-WheatGrain dataset/test/21/23-552-21-10.jpg]

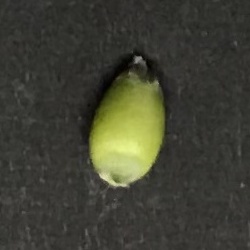

Supplement: Supplementary file 1 [file plants-12-04043-s001.zip › Figure S1-WheatGrain dataset/test/21/24-553-21-16.jpg]

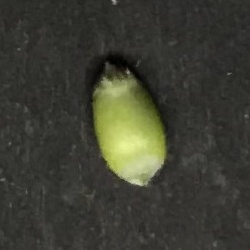

Supplement: Supplementary file 1 [file plants-12-04043-s001.zip › Figure S1-WheatGrain dataset/test/21/25-581-21-15.jpg]

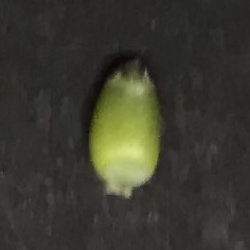

Supplement: Supplementary file 1 [file plants-12-04043-s001.zip › Figure S1-WheatGrain dataset/test/21/26-582-21-15.jpg]

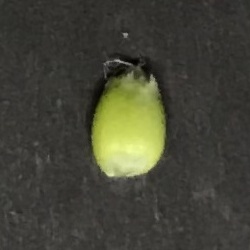

Supplement: Supplementary file 1 [file plants-12-04043-s001.zip › Figure S1-WheatGrain dataset/test/21/27-583-21-1.jpg]

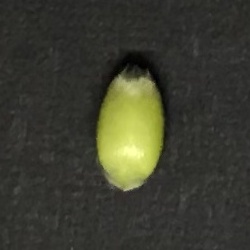

Supplement: Supplementary file 1 [file plants-12-04043-s001.zip › Figure S1-WheatGrain dataset/test/21/3-203-21-12.jpg]

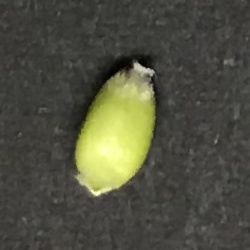

Supplement: Supplementary file 1 [file plants-12-04043-s001.zip › Figure S1-WheatGrain dataset/test/21/4-251-21-18.jpg]
