# Supplementary material for: Comparison of Different Machine Learning Algorithms for the Prediction of the Wheat Grain Filling Stage Using RGB Images
Source: Plants (Basel). 2023 Nov 30;12(23):4043. doi: 10.3390/plants12234043 (PMC10708398; doi:10.3390/plants12234043)
Supplement: Supplementary file 1 [file plants-12-04043-s001.zip › plants-2726337-supplementary Tables.pdf]

Table S1. Details of datasets.

DAA (days after anthesis)	Number of samples	Samples
6	516	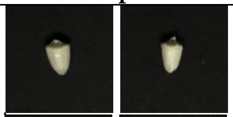
9	539	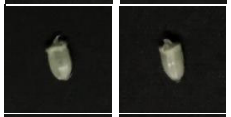
12	538	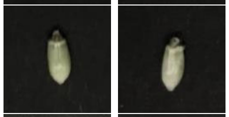
15	539	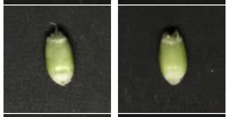
18	499	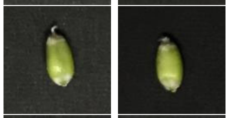
21	499	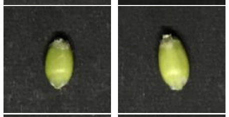
24	539	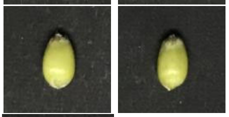
27	521	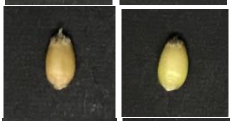
30	519	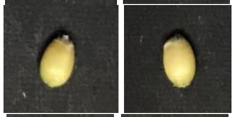
33	532	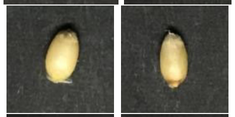
36	539	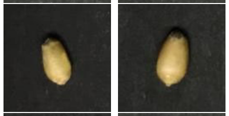
39	479	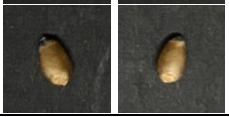

Table S2. Model size.

Algorithm		Model size (MB)
Traditional machine learning	Decision Tree	0.01
	Support Vector Machine	1.01
	Random Forest	0.15
Deep learning	HRnet18	74.37
	Densenet121	27.15
	Inception v3	83.51
	VGG16	512.36
	Resnet18	42.73
	Resnet50	90.07
	Resnet101	162.82
	VIT	146.01
Few-shot learning	/	72.19
